# Association between waist circumference and female infertility in the United States

**DOI:** 10.1371/journal.pone.0295360

**Published:** 2023-12-20

**Authors:** Jierong Ke, Yuntian Feng, Zhiyun Chen

**Affiliations:** 1 Center for Reproductive Medicine, Huizhou Central People’s Hospital, Huizhou, Guangdong, China; 2 Department of Medical Oncology, Huizhou First Hospital, Huizhou, Guangdong, China; Nazarbayev University School of Medicine, KAZAKHSTAN

## Abstract

**Background:**

Obesity has significant implications for fertility and reproductive health. However, evidences linking abdominal obesity to female infertility were limited and inconclusive. Our objective was to figure out the potential relationship between waist circumference (WC) and infertility among women of childbearing age in the United States using data from the National Health and Nutrition Examination Survey (NHANES).

**Methods:**

Our cross-sectional study included 3239 female participants aged 18–45 years. To explore the independent relationship between WC and female infertility, the weighted multivariable logistic regression and smoothed curve fitting were performed. Interaction and subgroup analyzes were then conducted for secondary analysis.

**Results:**

WC was positively associated with female infertility independent of BMI after adjusting for BMI and other potential confounders. In fully adjusted model, for every 1cm increase in waist circumference, the risk of infertility increased by 3% (OR = 1.03, 95% CI: 1.01–1.06). When WC was divided into five equal groups, women in the highest quintile had 2.64 times risk of infertility than that in the lowest quintile (OR = 2.64, 95% CI: 1.31–5.30). Smooth curve fitting revealed a non-linear but positively dose-dependent relationship between WC and female infertility. Furthermore, we found an inverted U-shaped relationship (turning point: 113.5 cm) between WC and female infertility in participants who had moderate recreational activities and a J-shaped relationship (turning point: 103 cm) between WC and female infertility in participants who had deficient recreational activities.

**Conclusions:**

Waist circumference is a positive predictor of female infertility, independent of BMI. Moderate recreational activities can lower the risk of female infertility associated with abdominal obesity.

## Introduction

Infertility is the medical condition in which pregnancy cannot be achieved through regular, unprotected sexual intercourse after attempting for a year or longer. It is a serious medical concern affecting about 8–12% of childbearing couples around the world [1]. In developed countries approximately 1 in 7 couples and in developing countries about 1 in 4 couples struggle with infertility [2]. In the United States, approximately 6.1 million women, accounting for 10% of the reproductive-age population, suffer from infertility [3]. Infertility is not only a serious psychological, social and economic burden for patients [4–6], but also increases the risk of reproductive cancers and metabolic diseases [7, 8].

Female infertility is a multifactorial reproductive disorder including ovulatory dysfunction, tubal obstruction, cervical factors, endometriosis, diminished ovarian reserve, uterine pathologies, or unexplained infertility [9] that can be triggered by genetic, infectious, environmental or lifestyle factors [5]. Lifestyle habits including obesity, have a negative impact on reproductive health [10].

Obesity is an increasing global health concern with significant implications for fertility and reproductive health [11]. From 2007 to 2016, the rate of obesity in US women older than 20 years increases from 35.4% to 41.1% and the incidence of severe obesity grows from 7.3% to 9.7% [12]. Previous researches provided evidences of a positive association between BMI and infertility [13–16]. However, the connections also prove to be inconclusive [17, 18]. Furthermore, as a marker of adiposity, BMI is thought to be deficient for its missing of measuring body fat distribution or fat mass [19, 20]. In addition, BMI may misclassify muscular individuals as obese [21]. For these reasons, it may be inappropriate to use BMI alone for obesity assessment. In the past decades, other markers for evaluating obesity have attracted lots of interest.

Waist circumference, which is an easily measured anthropometric parameter, is correlated with fat mass and has been proposed to classify central obesity [22]. In fact, several studies have revealed that WC alone or in conjunction with BMI is related to the risk of multiple chronic diseases or assisted reproductive technology outcomes [23–27]. However, previous researches regarding the association between WC and female infertility are very limited. Thus, we aim to identify the potential association between WC and infertility among women of childbearing age in the United States using a national, population-based sample.

## Methods

### Data sources

The data from NHANES were used to explore the association of WC with female infertility and detailed information on data collection and methodology was publicly accessible [28]. NHANES is a cross-sectional survey designed to collect information about the health and nutritional status of the civilian, non-institutionalized population of the United States through interviews or physical examinations. Every two years, NHANES investigates a representative sample of about 10,000 persons across the country, providing valuable data for researchers around the world. The Ethics Committee of Huizhou Central People’s Hospital reviewed and approved our study (No. 20220501016). The protocols of NHANES were approved by the Ethics Review Board of American National Center for Health Statistics (NCHS). Written informed consent form was signed by each participant (with witness if required) when they were recruited to NHANES. NHANES data are publicly available and anonymized for researchers. In our study the informed consent was waived as our study was the secondary analysis of NHANES data and we didn’t have access to information that could identify individual participants during or after data collection.

### Study population

**Fig 1:**
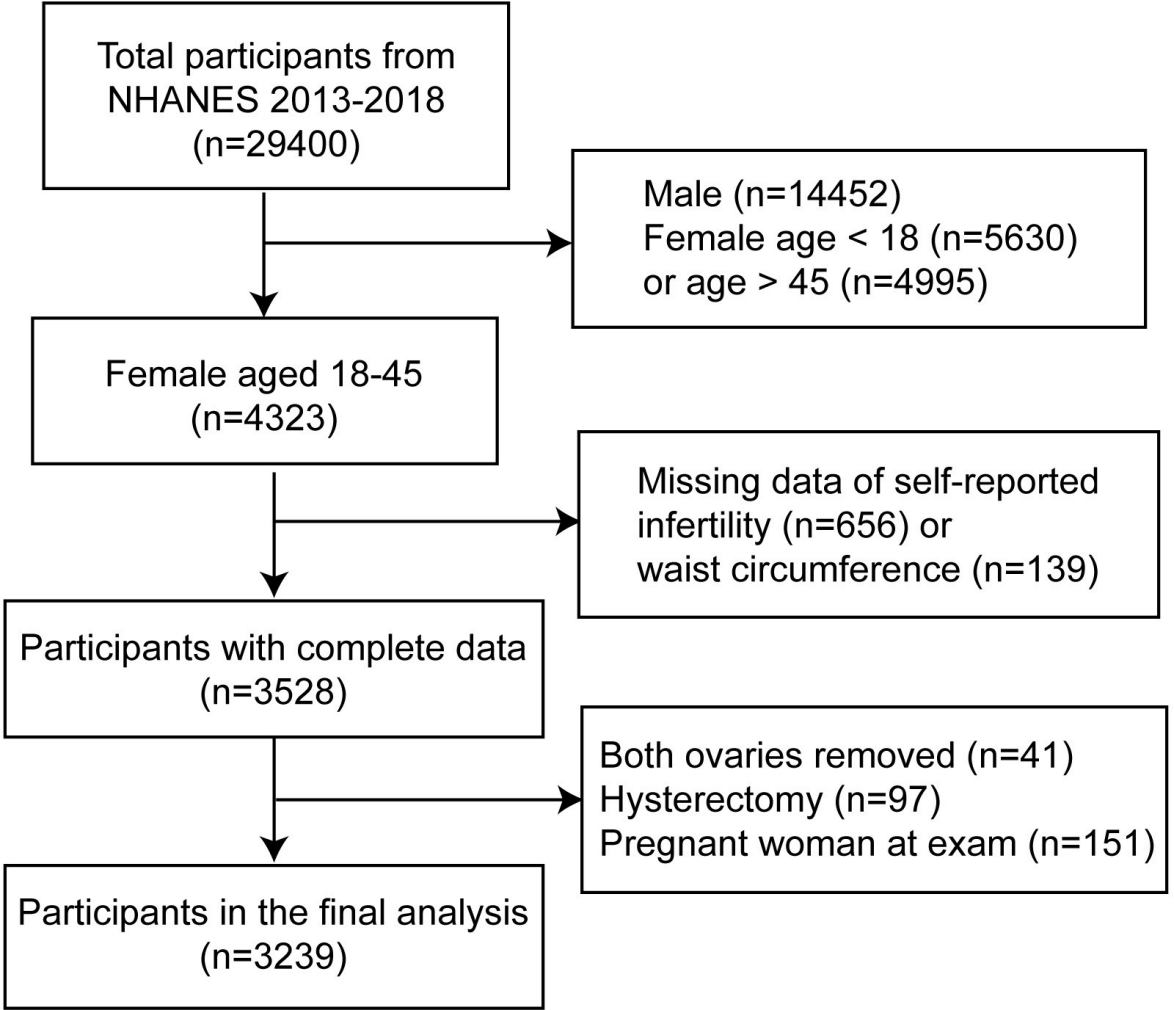
Flow chart of sample selection.

In our study, three two-year cycles of NHANES data (participants were recruited in 2013–2014, 2015–2016, and 2017–2018) were selected for analysis because only the data during these periods contained the reproductive health questionnaires that addressed our issues. Our study included female participants aged 18–45 years with complete data of independent and dependent variables. Women who were pregnant or anatomically unable to become pregnant would be excluded. Initially, 29400 participants were included in our study. We excluded female participants younger than 18 years (n = 5630) or older than 45 years (n = 4995) and 14452 male participants. Participants with missing data of self-reported infertility (n = 656) and WC (n = 139) were excluded. Participants who had both ovaries removed (n = 41), had a hysterectomy (n = 97) or were pregnant at exam (n = 151) were also excluded. Finally, our study included 3239 subjects for analysis (Fig 1).

### Variables

In our analysis, the independent variable was WC, which was measured by trained health technicians as previously described [29]. The dependent variable of interest was self-reported female infertility which was obtained from the questionnaire of reproductive health. The questions in this questionnaire were asked by trained interviewers utilizing MEC’s computer- assisted personal interview system. Participants were asked about their pregnancy history with the question of “Have you ever attempted to become pregnant over a period of at least a year without becoming pregnant?” Women who responded affirmatively were classified as infertility, while those who answered “no” were considered as the opposite category.

In addition, the variates presented in Table 1 were included as covariates according to previous described or clinical experience [18, 30]. Demographic characteristics included age, race/ethnicity, education level, marital status, and poverty-income ratio (PIR). Several lifestyle covariates were included, such as recreational activities, smoked at least 100 cigarettes in life, and drinking (based on the question "How often drink alcohol over past 12 months"). Health variables included hypertension and diabetes mellitus. The anthropometric covariate (BMI) and reproductive factor (ever been pregnant) were also included. All detailed data acquisition processes and measurement procedures of above variates were available on the NCHS website.

**Table 1:** The weighted characteristics of study population.

Variables	Total (n = 3239)	Non-infertility (n = 2912)	Infertility (n = 327)	P-value
**Age (years)**	31.2 ± 0.2	30.7 ± 0.2	35.1 ± 0.5	<0.0001
**Race/ethnicity (%)**				0.5258
Mexican American	12.3	12.3	11.9	
Other Hispanic	7.9	8.2	6.2	
Non-Hispanic White	55.8	55.2	60	
Non-Hispanic Black	13.3	13.4	12.9	
Other Race	10.7	10.9	9.0	
**Education (%)**				0.0001
Less than high school	10.1	10	10.6	
High school	18.2	17.9	20.8	
More than high school	64.8	64.4	68	
Not recorded	6.9	7.7	0.7	
**Marital status (%)**				<0.0001
Married or Living with partner	55.0	52.5	75.3	
Live alone	38.1	39.8	24.0	
Not recorded	6.9	7.7	0.7	
**PIR**	2.6 ± 0.1	2.6 ± 0.1	2.8 ± 0.1	0.1340
**Body mass index (**kg/m^2^**)**	29.0 ± 0.2	28.7 ± 0.2	32.0 ± 0.7	0.0002
**Waist circumference (cm)**	94.7 ± 0.5	93.7 ± 0.5	102.8 ± 1.6	<0.0001
**Ever been pregnant (%)**				<0.0001
Yes	62.4	59.7	84.6	
No	30.6	32.5	14.6	
Not recorded	7.0	7.8	0.8	
**Recreational activities (%)**				0.1192
Deficient	39.5	38.8	44.8	
Moderate	23.8	23.7	24.7	
Vigorous	36.8	37.5	30.5	
**Smoked ≥100 cigarettes in life (%)**				0.0049
Yes	29.8	28.9	37.4	
No	70.2	71.1	62.6	
**Drinking (%)**				0.0118
Yes	77.8	77.8	77.2	
No	7.6	7.1	12.4	
Not recorded	14.6	15.1	10.4	
**Hypertension (%)**				0.0194
Yes	12.3	11.6	18.7	
No	87.6	88.4	81.3	
Not recorded	0.1	0.1		
**Diabetes Mellitus (%)**				0.0008
Yes	2.8	2.3	6.7	
No	95.6	96.2	90.7	
Borderline	1.5	1.4	2.5	
Not recorded	0.1	0.1		

Survey-weighted mean ± SE for continuous variables: P-value was by survey-weighted linear regression.

Survey-weighted percentage for categorical variables: P-value was by survey-weighted Chi-square test.

### Statistical analysis

Statistical analysis was carried out using Empower Stats software (X&Y Solutions Inc., Boston, USA) and the R package (version 3.4.3). Continuous variables were presented as mean ± standard error (SE), while categorical variables were expressed as percentages. Missing values of BMI (n = 4) and PIR (n = 263) were estimated by mean value. Missing data for categorical variates were taken as a group. The 6-year sampling weight of three two-year cycles used in our analyses was obtained by dividing the combined two-year weight (WTMEC2YR) by three. Considering the complex sampling design of NHANES, the sampling parameters (weight, strata and clusters) was taken into account in the analysis of the association between WC and female infertility. We performed weighted linear regression to analyze the characteristics of study population for continuous variables, while weighted chi-square tests were used for categorical variables. The weighted multivariable logistic regression models were implemented to explore the independent association between WC and female infertility after adjusting for potential confounders. Four statistical models were constructed: model 1, unadjusted model; model 2, adjusted for age, race and education level; model 3, adjusted for age, race and education level and BMI; model 4, adjusted for all covariates listed in Table 1. For sensitivity analyses, we checked the association between WC as a categorical variable (WC quintiles: Q1: 56.4–77.8 cm; Q2: 77.9–86.4 cm; Q3: 86.5–96.0 cm; Q4: 96.1–109.2 cm; Q5: 109.3–172.5 cm) and infertility to confirm the results of WC as a continuous variable. In addition, we used a weighted smoothed curve fitting and a generalized additive model to reveal the linearity or non-linearity associated between WC and infertility. Subsequently, interaction and subgroup analysis were then performed after adjusting for the covariates except for the stratification factor itself. We also performed weighted smoothed curve fittings to reveal the non-linearity of WC and female infertility in the subgroup analyses and the two-piecewise linear regression model was carried out to investigate the saturation or threshold effect of WC on the risk of infertility in the subgroup analyses. Statistical significance was defined at a level of p < 0.05.

## Results

### Population characteristics

The weighted characteristics of the participants aged 18 to 45 years were presented in Table 1 according to their fertility status. Of the 3239 individuals, 327 (11.1%) women were infertile. Compared to the group without infertility, infertile women were older (35.1 vs. 30.7). They had a higher WC (102.8 cm vs. 93.7 cm) and a higher BMI (32.0 vs. 28.7). They were more likely to have better education (more than high school: 68% vs. 64.4%), to have a regular partner (75.3% vs. 52.5%), to have ever been pregnant (84.6% vs. 59.7%), to have hypertension (18.7% vs. 11.6%) and diabetes mellitus (6.7% vs. 2.3%). They were more likely to be smokers (37.4% vs. 28.9%), but not drinkers (77.2% vs. 77.8%). There were no statistical differences in race, PIR and recreational activities.

### Association between WC and female infertility

Four weighted univariate or multivariate logistic regression models were constructed to examine the association between WC and female infertility (Table 2). Covariates were included according to previous described or clinical experience [18, 30]. In model 1 and model 2, a positive association was observed with each additional unit (cm) of WC leading to a 2% increase in infertility risk (Table 2). Furthermore, the positive association became stronger in model 3 and model 4 (model 3: OR = 1.04, 95% CI: 1.02–1.06; model 4: OR = 1.03, 95% CI: 1.01–1.06).

**Table 2:** Association between waist circumference (cm) and female infertility.

	Model 1 OR (95%CI)	Model 2 OR (95%CI)	Model 3 OR (95%CI)	Model 4 OR (95%CI)
WC (cm)	1.02 (1.02,1.03)***	1.02 (1.01,1.03)***	1.04 (1.02, 1.06)***	1.03 (1.01, 1.06)**
**Quintiles of WC (cm)**				
Q1 (56.4–77.8)	Reference	Reference	Reference	Reference
Q2 (77.9–86.4)	1.83 (1.08, 3.11)*	1.41 (0.83, 2.38)	1.41 (0.84,2.37)	1.33 (0.77, 2.29)
Q3 (86.5–96.0)	2.21 (1.31, 3.72)**	1.63 (0.94, 2.84)	1.64 (0.91, 2.94)	1.50 (0.81, 2.77)
Q4 (96.1–109.2)	2.67 (1.59, 4.47)***	1.82 (1.07, 3.09)*	1.82 (1.06, 3.13)*	1.66 (0.95, 2.90)
Q5 (109.3–172.5)	4.46 (2.75, 7.24)***	3.15(1.87, 5.32)***	3.16 (1.61, 6.22)**	2.64 (1.31, 5.30)*
P for trend	<0.0001	0.0003	0.009	0.0375
**Stratified by BMI (kg/m** ^**2**^ **)**				
BMI≤24.9	1.08 (1.04, 1.12)***	1.05 (1.02, 1.09)**	1.05 (1.02, 1.09)**	1.05 (1.01, 1.09)*
24.9<BMI≤29.9	1.02 (0.98, 1.07)	1.01 (0.96, 1.05)	1.00 (0.96, 1.04)	1.00 (0.96, 1.04)
BMI>29.9	1.02 (1.01, 1.03)**	1.02 (1.00, 1.03)*	1.02 (1.00, 1.03)*	1.02 (1.00, 1.03)*

Note: Model 1: no covariates were adjusted. Model 2: age, race/ethnicity and education level were adjusted; Model 3 age, race/ethnicity, education level and BMI were adjusted; Model 4: covariates listed in Table 1 were adjusted. In the subgroup analysis stratified by BMI, the models were not adjusted for BMI.

*P < 0.05

**P < 0.01

***P < 0.001

Since BMI was a strong confounder, we performed a subgroup analysis stratified by BMI (Table 2 and Fig 3). In fully adjusted model, the positive relationship between WC and infertility existed in groups of BMI ≤ 24.9 kg/m2 (OR = 1.05, 95% CI: 1.01–1.09) and BMI > 29.9 kg/m2 (OR = 1.02, 95% CI: 1.00–1.03).

### Identification by sensitivity analysis

**Fig 2:**
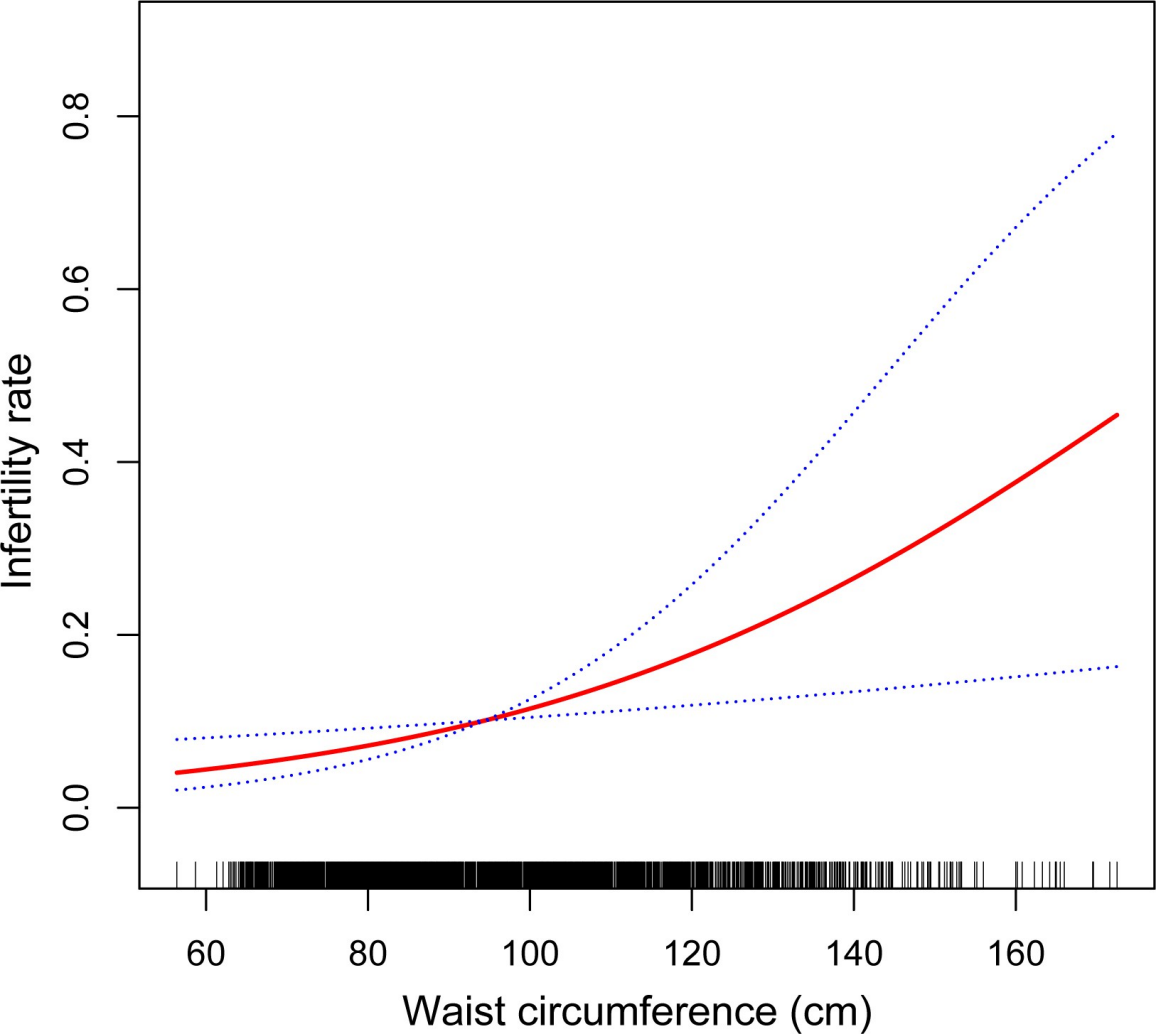
Association between WC and infertility rate (using generalized additive models). Covariates listed in Table 1 were adjusted.

We also conducted sensitivity analysis to confirm the robustness and accuracy of the results. First, WC was divided into five equal groups (Q1: 56.4–77.8 cm; Q2: 77.9–86.4 cm; Q3: 86.5–96.0 cm; Q4: 96.1–109.2 cm; Q5: 109.3–172.5 cm) and we found that the result of the categorical variable was consistent with the effect of WC as a continuous variable. The risk of infertility in the highest quintile was 2.64 times than that in the lowest quintile in the fully adjusted model (Table 2; OR = 2.64, 95% CI: 1.31–5.30, P for trend < 0.05). In addition, we performed an adjusted smoothed curve fitting and a generalized additive model to characterize the potential non-linear relationship between WC and female infertility based on the fully adjusted model. The results showed that the association between WC and female infertility was non-linear but positively dose-dependent (Fig 2).

### Subgroup analysis

**Fig 3:**
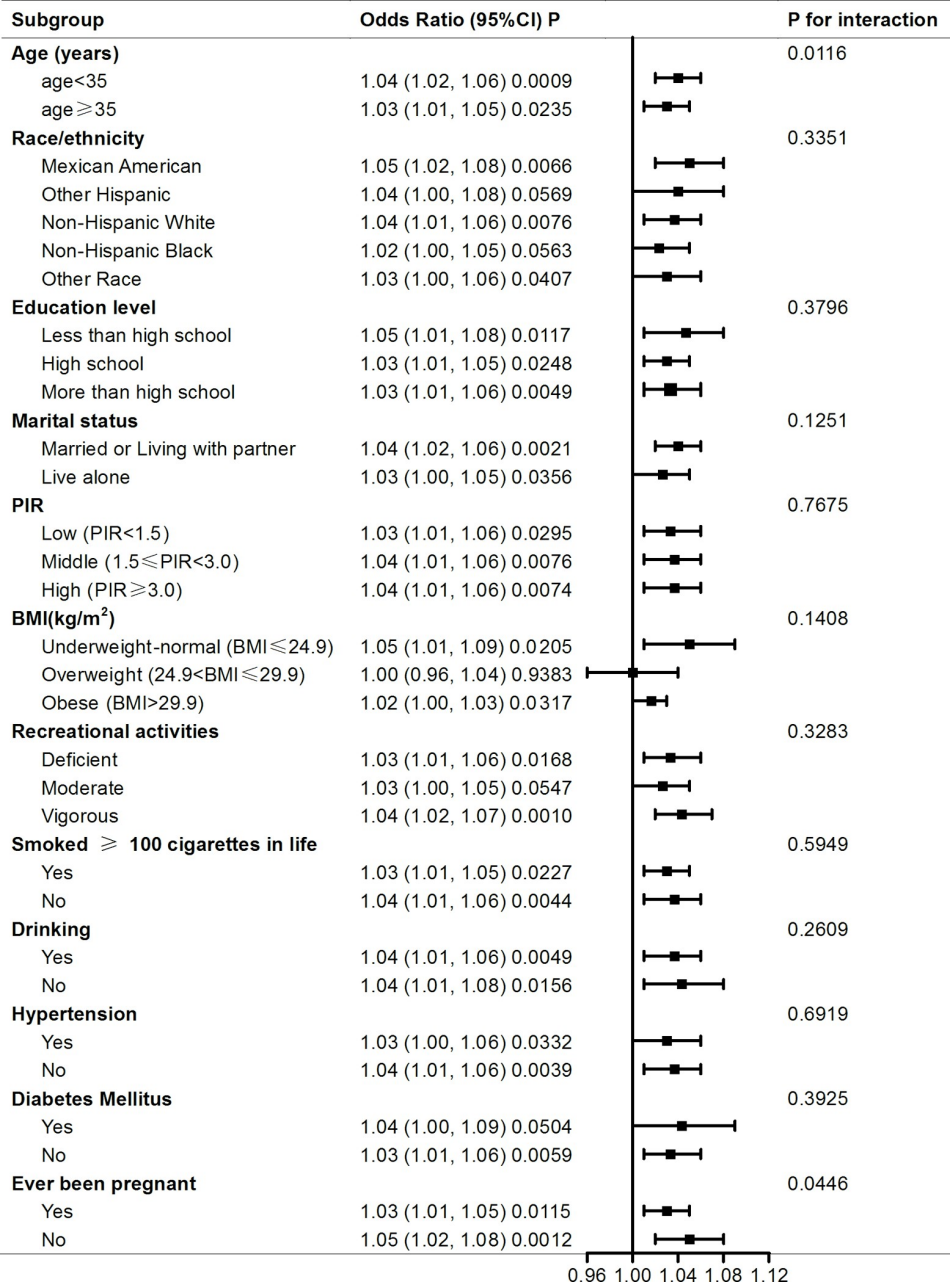
Subgroup analysis for the association between WC and female infertility. Each stratification was adjusted for covariates listed in Table 1 except for the stratifying variable itself.

**Fig 4:**
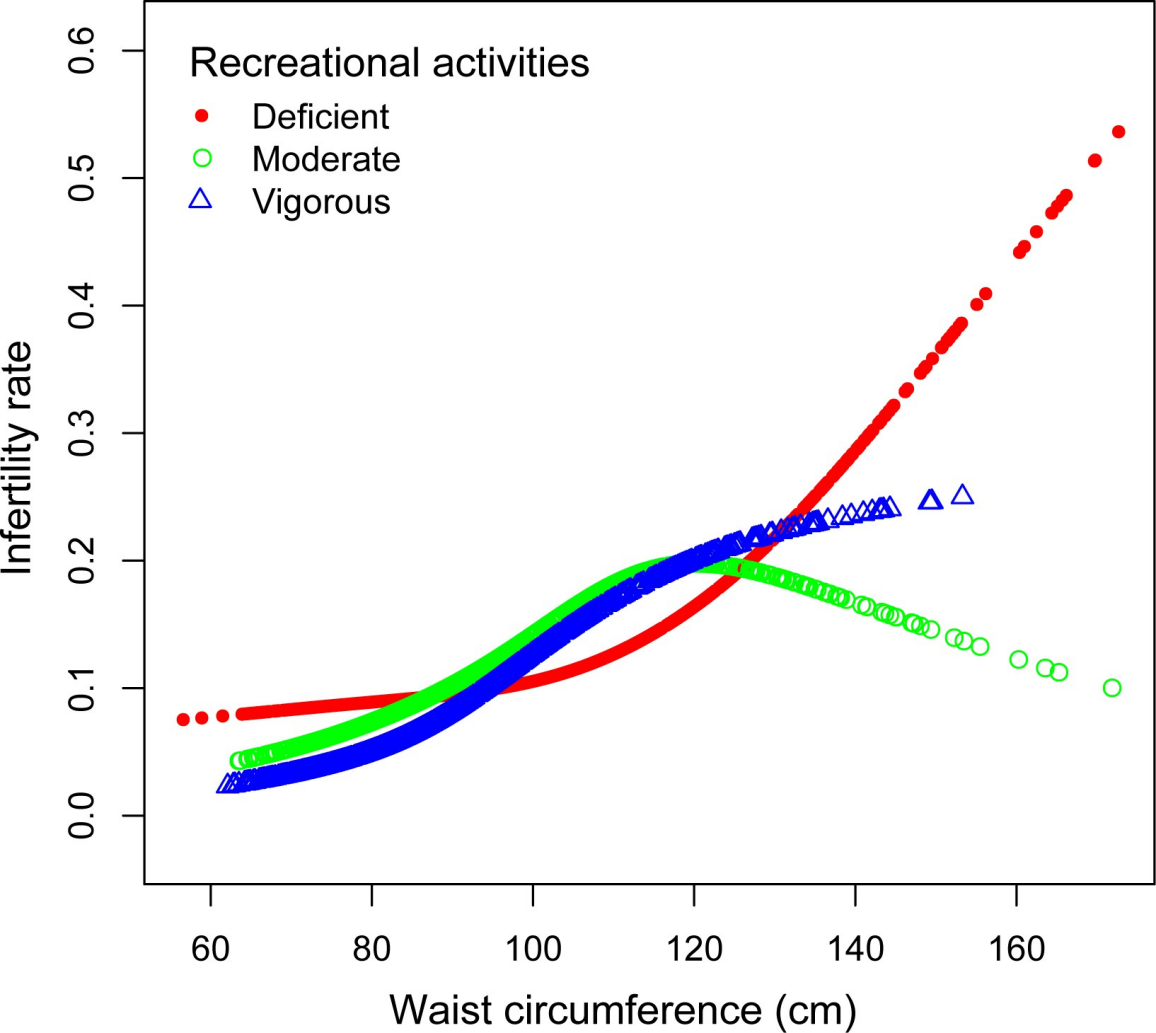
Association between WC and infertility stratified by recreational activities. Covariates listed in Table 1 were adjusted except for recreational activities.

To examine the consistency of the correlation between WC and female infertility across subgroups, we performed interaction and subgroup analyzes stratified by various variables, as presented in Fig 3. We found that this association was consistent across subgroups of race, education level, marital status, PIR, BMI, recreational activities, smoking, alcohol consumption, hypertension and diabetes mellitus (Fig 3; all P for interaction > 0.05). In addition, we found that variables of age and ever been pregnant may have interactions with WC associated with infertility (P for interaction < 0.05, Fig 3). Moreover, we evaluated the influence of several lifestyle habits on the association between WC and female infertility through adjusted smoothed curve fitting stratified by smoking, drinking and recreational activities to provide reference for fertility. The trend of the smoothed curves was consistent in most subgroups (S1 Fig), but not in recreational activity (Fig 4). We found an inverted U-shaped relationship between WC and female infertility among participants with moderate recreational activities, and a J-shaped relationship between WC and female infertility among participants with deficient recreational activity (Fig 4). The two-piecewise linear regression model revealed that there was a inflection point at a WC of 113.5 cm in subgroup of having moderate recreational activities and a inflection point at a WC of 103 cm in subgroup of deficient recreational activities (Table 3). For groups of deficient and vigorous recreational activities, there was positive association between WC and female infertility (Fig 3). For participants having moderate recreational activities, the positive association between WC and female infertility became irrelevant (Fig 3, OR = 1.03, 95% CI: 1.00–1.05, P value = 0.0547).

**Table 3:** Saturation or threshold effect analysis of waist circumference on female infertility stratified by moderate recreational activities using a two-piecewise linear regression model.

Outcome:	Recreational activities (OR (95%CI) P value)		
	Deficient	Moderate	Vigorous
Fitting by standard linear model	1.01 (0.99, 1.04) 0.3309	1.02 (0.98, 1.06) 0.3490	1.06 (1.01, 1.10) 0.0081
Fitting by two-piecewise linear model			
Inflection point (K, cm)	103	113.5	101.7
waist circumference < K	0.99 (0.96, 1.02) 0.6420	1.04 (0.99, 1.08) 0.1014	1.08 (1.03, 1.13) 0.0016
waist circumference > K	1.03 (1.00, 1.06) 0.0458	0.97 (0.91, 1.03) 0.2574	1.03 (0.98, 1.08) 0.2686
Log-likelihood ratio	0.013	0.008	0.075

Note: Covariates listed in Table 1 were adjusted except for recreational activities.

## Discussion

The national data from NHANES 2013–2018 was used to examine the correlation between WC and female infertility in the United States. And we found a positive association between WC, as a clinical parameter of abdominal obesity, and infertility independent of BMI after adjusting for confounding factors. Our results showed that this relationship was non-linear but positively dose-dependent. Furthermore, we found an inverted U-shaped relationship (turning point: 113.5 cm) between WC and female infertility in participants who had moderate recreational activities and a J-shaped relationship (turning point: 103 cm) between WC and female infertility in participants who had deficient recreational activities.

Obesity and infertility are both growing health concern worldwide. The high prevalence of these two diseases necessitates a more comprehensive understanding of their interrelationship. Previous studies have focused on the association between general obesity, defined by BMI, and infertility [13–16, 18]. To date, evidences linking abdominal obesity to female infertility are still scarce and remain controversial. In a prospective cohort study of 264 infertile women from Massachusetts General Hospital, Li et al. reported a negative association between WC and the probability of live birth in infertile women undergoing assisted reproductive technology (ART), independent of BMI [27]. In a pilot study conducted in Australia about the weight loss and ART outcomes in obese infertile women, Moran et al. found that a reduction in WC was associated with an increased likelihood of pregnancy, while changes in BMI did not predict ART outcomes [31]. In a cross-sectional observational study of 788 infertile women in Brazil, Christofolini and colleagues observed that body fat distribution, particularly WC, was more relevant than BMI in predicting ART outcomes [26]. Another observational study from the Netherlands revealed that abdominal subcutaneous fat and not the intraabdominal fat compartment was associated with anovulation in women with obesity and infertility [32]. However, in secondary analyzes of a prospective, randomized, and multicenter clinical trial of women undergoing intrauterine insemination, Hansen et al. reported that neither BMI nor WC were associated with the outcome of live birth [33]. These inconsistent conclusions may be related to the study design, study population, methodological differences in the variables, or the control for confounding variables. In the present study, using a nationally representative sample, our study supported the association between increased waist circumference and impaired fertility in US women of childbearing age. As far as we know, our study was the first epidemiological investigation of the association between WC and female infertility.

The mechanisms underlying the deleterious effects of obesity on reproductive health may be complex and multifactorial. Previous researches have indicated that obesity is associated with alterations in hormonal profiles, including excessive secretion of leptin, insulin, adipokine, androgen and estrogen, and decreased levels of adiponectin and sex hormone binding globulin, which can lead to functional alteration of the hypothalamus -pituitary-ovarian axis, menstrual disorder and impairs folliculogenesis [11, 34–37]. Insulin resistance, a hallmark of obesity, has also been implicated in the pathogenesis of female infertility (especially in the pathogenesis of the polycystic ovarian syndrome), as it can exacerbate hormonal imbalance and ovulation disorder [38–40]. Chronic inflammation and oxidative stress, commonly seen in obese individuals, may further exacerbate these effects by promoting insulin resistance, disrupting ovarian function, and impairing embryo implantation [41, 42]. Despite these possibilities, further researches are needed to elucidate the underlying molecular mechanism for the association between WC and infertility, and to develop effective interventions to prevent and treat obesity-related infertility.

To assess the consistency of the association between WC and female infertility across various subgroups, we conducted subgroup analyses. Then we found an inverted U-shaped relationship (turning point: 113.5 cm) between WC and female infertility in participants who had moderate recreational activities and a J-shaped relationship (turning point: 103 cm) between WC and female infertility in participants who had deficient recreational activities. Our study showed the protective effect of moderate recreational activities against the risk of infertility associated with WC, especially when WC was bigger than 113.5 cm. Previous study also showed that moderate to high levels of physical activity can significantly decrease the likelihood of infertility and that this level of physical activity was a common protective factor [43]. A prospective cohort study of 3,628 women in Danish showed a weak positive correlation between moderate physical activity and fertility independent of BMI and an inverse association between vigorous physical activity and time to pregnancy in all subgroups of women excepted for overweight and obese women. These results indicated that moderate or vigorous physical activity could improve fertility in overweight and obese women [44]. Physical activity had also been shown to reduce oxidative damage and pro-inflammatory status [45], which may contribute to the improvement in fertility [41, 42]. Our results supported that moderate recreational activity can reduce the risk of infertility associated with increased waist circumference, but insufficient or vigorous recreational activity may impair conception. Therefore, women trying to conceive may increase their chances of getting pregnant by monitoring their waist circumference and engaging in moderate recreational activities.

In the present research, we examined the relationship between WC and female infertility utilizing a national population-based sample. Furthermore, the inclusion of a sizable sample size in this study ensured robust statistical power. Nevertheless, some limitations should be acknowledged. First, the cross-sectional design of NHANES prevented us from establishing a causal relationship between waist circumference and female infertility. Second, since infertility was measured using self-reported data, participants might have difficulty in recalling the exact duration of their attempts to conceive. Third, although we controlled for potential covariates in this study, bias caused by residual confounding factor may also exist. Fourth, our results were limited to the United States and cannot be generalized to other countries. Fifth, missing values were usually informative and can affect the mean value if they were actually reported. Handling missing data by imputing the mean value may lead to bias of sample mean and underestimate of the degree of variation and thus affected the subsequent analyses. Because the number of missing values in our study was small, we handled the missing data of BMI (0.1%, n = 4) and PIR (8.1%, n = 263) by imputing the mean value even though this was generally not the best method. Therefore, further prospective studies with large samples and complete data may be necessary to overcome some of these limitations.

In conclusion, we demonstrated that WC was positively and dose-dependently associated with female infertility in the United States, independent of BMI. Moreover, we found an inverted U-shaped relationship (turning point: 113.5 cm) between WC and female infertility in participants who had moderate recreational activities and a J-shaped relationship (turning point: 103 cm) between WC and female infertility in participants who had deficient recreational activities. Our results indicated that effective waist circumference management strategies and moderate recreational activities were required to reduce the risk of abdominal obesity and improve reproductive health.

## Supporting information

S1 Fig Association between WC and female infertility rate stratified by covariates (using generalized additive models). Each stratification was adjusted for covariates listed in Table 1 except for the stratifying variable itself.
(TIF)
