# Supplementary figures and images for: Association between waist circumference and female infertility in the United States
Source: PLoS One. 2023 Dec 20;18(12):e0295360. doi: 10.1371/journal.pone.0295360 (PMC10732459; doi:10.1371/journal.pone.0295360)

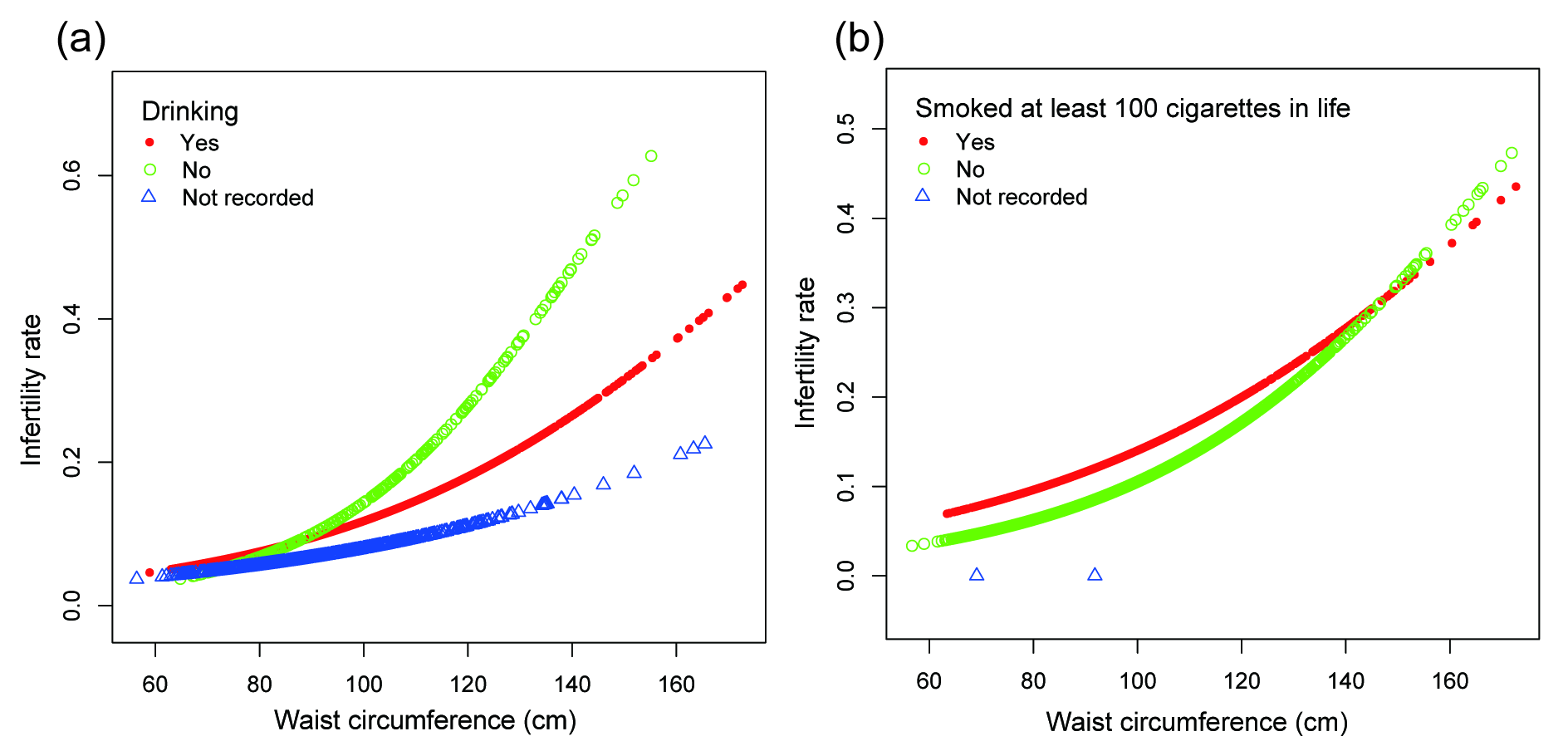

Supplement: S1 Fig — Each stratification was adjusted for covariates listed in Table 1 except for the stratifying variable itself. (TIF) [file pone.0295360.s001.tif]
